# Modern Dietetics

**Published:** 1906-04-28

**Authors:** 


### MODERN DIETETICS.

The revised edition of Dr. Robert Hutchison's book [1] is a mine of information laboriously collated. This country has been rather behindhand in the scientific attack upon the problems of diet, and as a natural consequence the amateur has to some extent appropriated the field. This being the case, it is clearly the business of the faculty to accumulate such a knowledge of the subject—an important one as everyone will admit—as will enable him to speak with some assurance to the faddist at the gate. It is rather unfortunate for the author that Chittenden's disturbing studies should synchronise so closely with the appearance of the revised edition of his book, for Chittenden has somewhat shaken many cherished beliefs as to the quantity of food-stuffs of various denominations necessary for the maintenance of health and activity. Nevertheless the book before us contains a mass of detailed information upon many matters not likely to be disturbed by the acceptance of Chittenden's results and not a little curious knowledge. We wonder, for instance, how many people outside the culinary line of business are aware that the green turtle-fat so prized at aldermanic entertainments is not fat at all, but a species of gelatin derived from the softer parts of the shield; or that it would be necessary to consume 700 walnuts in order to obtain the necessary amount of proteid required by the body every day: or, again, that a cocoanut weighing 1¼ lbs. contains ¼ lb. of fat, and that, since its price is about twopence, it is equivalent to a ¼ lb. of butter at 8d. a pound. By dint of such light sallies Dr. Hutchison has succeeded in reducing the heaviness of a work containing a massive array of facts, and we are grateful.

But there is one important matter in which Dr. Hutchison's knowledge has been productive of obscurity—we mean in the matter of mushrooms and other edible fungi. In the section devoted to this subject the common mushroom, the agaricus campestris, receives nothing but the barest acknowledgment, and no instructions are given for its identification. Dr. Hutchison forgets that he is not writing for botanists who can confidently distinguish a boletus from an agaricus, but for people who in the absence of a description of the common mushroom are likely to be confused by the warning "All those that are red or pink or orange or brown beneath the cap should be avoided." This direction is intended to apply to the boletus family alone, but the distinction is not sufficiently emphasised to serve the turn of the casual reader.

A section of the book is devoted to the discussion of vegetarianism. Dr. Hutchison is no vegetarian; he holds that man is eminently a mixed feeder, and that peoples who, being more or less strictly vegetarian, subsist upon a minimum of proteid, are lacking in energy. He points out that energy is not to be confused with muscular strength. "A grass-fed cart-horse is strong; a corn-fed hunter is energetic. Energy is a property of the nervous system; strength, of the muscles." There is much to support this thesis, but the question of vegetarianism has assumed an altered aspect since Chittenden's demonstration of the ease with which health and strength may be maintained upon a diet far less albuminous than that in common use.

The influence of alcohol upon the system commands a chapter to itself, as becomes the importance of the subject. The judicial finding of the author is that alcohol is an unnecessary article of diet in conditions of complete health, although within certain limits, say one to one and a-half fluid ounces of absolute alcohol or its equivalent per diem, it is not harmful, and may even be beneficial: that alcohol is not favourable to the production of sustained muscular effort, nor to the production of perfectly healthy brain work.

In addition to dealing with such matters of popular interest, the book contains a great number of useful analyses of patent and other food-stuffs, and is in every respect an agreeable addition to any library, whether technical or general.

[1] Food and the Principles of Dietetics. (Arnold. 16s. net.)

